# Imaging the transmembrane and transendothelial sodium gradients in gliomas

**DOI:** 10.1038/s41598-021-85925-9

**Published:** 2021-03-23

**Authors:** Muhammad H. Khan, John J. Walsh, Jelena M. Mihailović, Sandeep K. Mishra, Daniel Coman, Fahmeed Hyder

**Affiliations:** 1 Department of Biomedical Engineering, Yale University, N143 TAC (MRRC), 300 Cedar Street, New Haven, CT 06520 USA; 2 Department of Radiology and Biomedical Imaging, Yale University, New Haven, CT 06520 USA

**Keywords:** Cancer imaging, Cancer metabolism, Cancer microenvironment, Tumour biomarkers, CNS cancer, Tumour heterogeneity, Blood-brain barrier

## Abstract

Under normal conditions, high sodium (Na^+^) in extracellular (Na^+^_e_) and blood (Na^+^_b_) compartments and low Na^+^ in intracellular milieu (Na^+^_i_) produce strong transmembrane (ΔNa^+^_mem_) and weak transendothelial (ΔNa^+^_end_) gradients respectively, and these manifest the cell membrane potential (*V*_*m*_) as well as blood–brain barrier (BBB) integrity. We developed a sodium (^23^Na) magnetic resonance spectroscopic imaging (MRSI) method using an intravenously-administered paramagnetic polyanionic agent to measure ΔNa^+^_mem_ and ΔNa^+^_end_. In vitro ^23^Na-MRSI established that the ^23^Na signal is intensely shifted by the agent compared to other biological factors (e.g., pH and temperature). In vivo ^23^Na-MRSI showed Na^+^_i_ remained unshifted and Na^+^_b_ was more shifted than Na^+^_e_, and these together revealed weakened ΔNa^+^_mem_ and enhanced ΔNa^+^_end_ in rat gliomas (vs. normal tissue). Compared to normal tissue, RG2 and U87 tumors maintained weakened ΔNa^+^_mem_ (i.e., depolarized *V*_*m*_) implying an aggressive state for proliferation, whereas RG2 tumors displayed elevated ∆Na^+^_end_ suggesting altered BBB integrity. We anticipate that ^23^Na-MRSI will allow biomedical explorations of perturbed Na^+^ homeostasis in vivo.

## Introduction

Sodium (Na^+^) concentration is normally low intracellularly (~ 10 mM) and high in blood and extracellular spaces (~ 150 mM)^1–3^, producing a strong transmembrane Na^+^ gradient (∆Na^+^_mem_ ≈ 140 mM) and a weak transendothelial Na^+^ gradient (∆Na^+^_end_ ≈ 0 mM). The ∆Na^+^_mem_ is coupled to the cell membrane potential (*V*_*m*_), nerve signaling^1^, muscle activity^4^ and osmoregulation^5^, while the ∆Na^+^_end_ impacts bicarbonate and proton transport between extracellular and intravascular compartments^2,6–8^ to signify blood–brain barrier (BBB) integrity^9,10^.

The sodium–potassium pump transports Na^+^ against its electrochemical gradient by consuming adenosine triphosphate generated through oxidative phosphorylation^11^. In glioblastoma (GBM), glycolysis is upregulated in relation to oxidative phosphorylation even with sufficient oxygen^12^. Aerobic glycolysis generates excessive amounts of hydrogen ions and lactate, which are extruded into the extracellular milieu, lowering the pH of the tumor microenvironment^13^. Since both the cell membrane and BBB regulate the ionic composition of the extracellular fluid^1,2^, we posited that maintaining ∆Na^+^_mem_ and ∆Na^+^_end_ becomes unsustainable in the tumor neurovascular unit. Activity of voltage-gated sodium channels on the cancer cell membrane helps regulate proliferation, migration, and invasion rather than excitability^14^. Similar to pH dysregulation in cancer, electrolyte imbalance also has a role in tumorigenesis^15,16^. Thus, being able to measure [Na^+^] across different compartments in vivo could be considered as an important biomarker.

Hyperpolarized *V*_*m*_ corresponds to quiescent cell cycle stages (*G*_*0*_ phase), and depolarized *V*_*m*_ indicates proliferative/replicative stages (*M* phase)^17–19^. Therefore, ∆Na^+^_mem_ is a biomarker for tumorigenicity and tumor aggressiveness. Determining [Na^+^] in the extracellular milieu usually involves inserting microelectrodes through the skull and reading voltage differences across cellular compartments^20^. In addition to issues of accurate microelectrode positioning and tissue penetration, such invasive techniques are challenging for human translation.

Angiogenesis is a crucial part of tumor growth^21^. Unlike normal tissues, the immature tumor vasculature exhibits saccular formations, hyperbranching, and twisted patterns that cause the BBB to be leaky. Prior cancer research avoided measuring [Na^+^] in blood presumably due to microhemorrhage concerns from ruptured blood vessels with microelectrodes. But given the gamut of anti-angiogenic therapies for GBM^22^, it is desirable to measure ∆Na^+^_end_ non-invasively.

Nuclear magnetic resonance (NMR) detects the isotope sodium-23 (^23^Na), a spin-3/2 quadrupolar nucleus. ^23^Na is 100% abundant and provides the second-strongest endogenous NMR signal in vivo, next to hydrogen (^1^H) which is a spin-1/2 non-quadrupolar nucleus^23^. ^23^Na magnetic resonance imaging (MRI) has greatly impacted stroke and ischemia research^24,25^, but reflects total sodium (Na^+^_T_)^26,27^ because ^23^Na-MRI signals from blood (Na^+^_b_), extracellular (Na^+^_e_), and intracellular (Na^+^_i_) compartments are difficult to separate. ^23^Na-MRI methods based on apparent diffusion coefficient (ADC), inversion recovery, and multiple quantum filtering (MQF) attempt to separate free (i.e., unbound or aqueous) and bound Na^+^ signals and their volume fractions, but suffer from low sensitivity. Moreover, diffusion methods necessitate large magnetic field gradients due to low gyromagnetic ratio (γ_Na_) and short longitudinal/transverse relaxation times (*T*_*1*_*/T*_*2*_) for ^23^Na. These ^23^Na-MRI methods are somewhat limited for probing the aqueous Na^+^_i_ signal because they cannot fully suppress major contributions from aqueous Na^+^_b_ and Na^+^_e,_ both of which dominate the Na^+^_T_ signal^26,28,29^. Thus, quantification of transmembrane (ΔNa^+^_mem_ = Na^+^_e_—Na^+^_i_) and transendothelial (ΔNa^+^_end_ = Na^+^_b_—Na^+^_e_) gradients has been challenging with ^23^Na-MRI. While detecting Na^+^_T_ is useful clinically, ∆Na^+^_end_ and ∆Na^+^_mem_ may help reveal relevant information about BBB viability and cellular proliferative/oncogenic potential in solid tumors.

Another approach to separate aqueous Na^+^ signals in vivo involves intravenous administration of an exogenous paramagnetic but polyanionic contrast agent (paraCA^*n-*^). The paraCA^*n-*^ consists of a lanthanide(III) cation core bound to an anionic macrocyclic chelate^30,31^. Since the paraCA^*n-*^ extravasates into extracellular space of most organs but does not enter cells, only Na^+^_e_ and Na^+^_b_ will be attracted to the paraCA^*n-*^ and experience a shift in the ^23^Na resonance frequency (depending on degree of paraCA^*n-*^ extravasation in each compartment) to separate the ^23^Na magnetic resonance spectroscopic imaging (MRSI) signals between Na^+^_b_, Na^+^_e_ and Na^+^_i_. While these paramagnetic effects will also shorten ^23^Na relaxation times slightly, the peak’s integral remains unaffected if data are acquired under fully relaxed conditions, and the peak’s shift can still be precise when there is marginal line broadening although integral bands will need to be widened so as to accurately estimate concentration. Proof-of-concept for this has been demonstrated in situ for the heart^32^ and liver^33^. Given the compromised BBB in tumors relative to healthy tissue, the ^23^Na-MRSI technique in conjunction with paraCA^*n-*^ is particularly efficacious in studying brain tumors.

**Figure 1:**
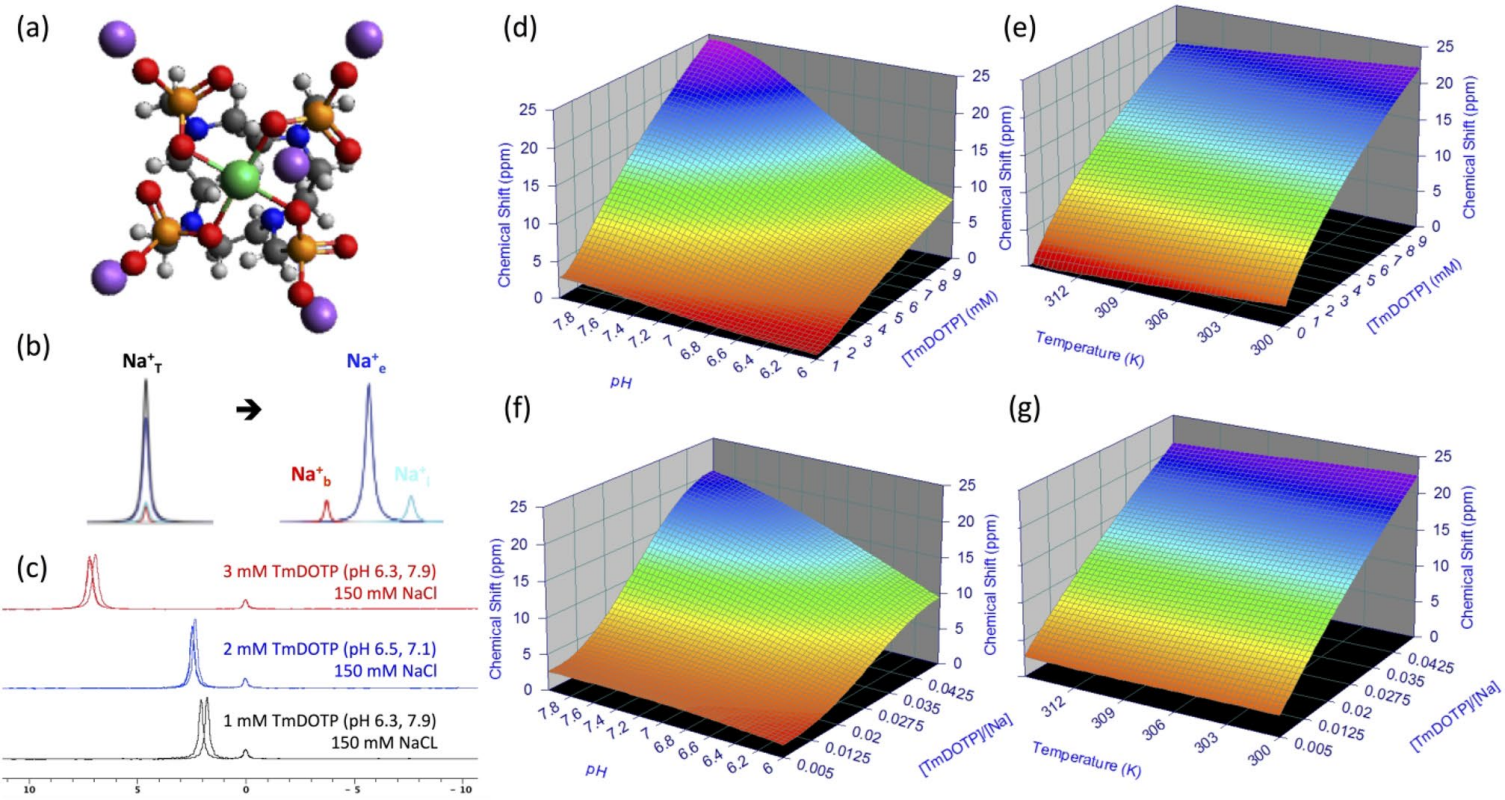
Shifting mechanism of the ^23^Na resonance in vitro. (**a**) Chemical structure of sodium thulium(III) 1,4,7,10-tetraazacyclododecane-1,4,7,10-tetrakis(methylenephosphonate) (Na_5_TmDOTP). The TmDOTP^5−^ complex consists of the Tm^3+^ ion chelated with DOTP^8−^. Each phosphonate-containing pendant arm on TmDOTP^5-^ has electron-donating groups on the oxygen atoms (red) to stabilize the Tm^3+^ conjugation with DOTP^8−^. The -5 charge simultaneously attracts five Na^+^ ions (purple), which experience a shift in the observed ^23^Na resonance that is dependent on [TmDOTP^5−^]. (**b**) In vivo, prior to TmDOTP^5−^ administration (left), the ^23^Na spectrum yields only a single peak representing the total sodium (Na^+^_T_) comprising blood (Na^+^_b_), extracellular (Na^+^_e_), and intracellular (Na^+^_i_ ) compartments. Following TmDOTP^5−^ administration (right), the peaks become spectroscopically separable based on [TmDOTP^5−^] in each compartment. Integrals of these peaks will be representative of aqueous [Na^+^] in each compartment. (**c**) A two-compartment coaxial cylinder tube setup was employed for in vitro observation of the chemical shift separation scheme (Figure S1). The inner tube (smaller volume) was filled with 150 mM NaCl, while the outer tube (larger volume) was filled with the same solution in addition to various amounts of TmDOTP^5−^, each subject to different pH conditions. Thus, all ^23^Na spectra from this phantom setup displayed a small unshifted peak from the inner compartment and a larger shifted peak. The outer-to-inner volume ratio was 8.6, explaining the difference in sizes of the peaks. Exemplary traces of ^23^Na spectra show that the shift is much more sensitive to [TmDOTP^5−^] (2.77 ppm/mM) than to variations in pH (0.25 ppm/pH unit) or temperature (0.03 ppm/°C). The downfield peaks in the red, blue, and/or black spectra are shifted differently due to varying TmDOTP^5−^ concentrations, and these shifts exceed those caused by pH, but all of these shifts are detectable independent of broadening caused by TmDOTP^5−^ (see also Figure S1). Plots (**d**,**e**) show that temperature, pH, and [TmDOTP^5−^] all contribute to variations of the ^23^Na chemical shift. However, these plots depict ranges of pH and temperature that are unlikely for in vivo settings (i.e., changes over 2 full pH units and temperature changes over 15 °C). Moreover, [Na^+^] in vivo (~ 150 mM in blood and extracellular space) is extremely high compared to [TmDOTP^5−^]. Therefore, variations in ^23^Na chemical shift are primarily dependent on [TmDOTP^5−^]/[Na^+^] thereby rendering (**f**) pH and (**g**) temperature dependencies negligible. Data points were fit to Chebyshev rational polynomials using TableCurve 3D v4.0.05 (Systat Software, San Jose, CA, USA; https://systatsoftware.com/products/tablecurve-3d/).

The most effective paraCA^*n-*^ for aqueous compartmental ^23^Na separation is^34^ the thulium(III) cation (Tm^3+^) complexed with 1,4,7,10-tetraazacyclododecane-1,4,7,10-tetrakis(methylenephosphonate) (DOTP^8-^) to form TmDOTP^5−^ (Fig. 1a). TmDOTP^5−^ has many applications in animal models, both with ^1^H-NMR^35–38^ and ^23^Na-NMR^33,39^, demonstrating that these Tm^3+^ agents are non-toxic at the doses presently being used. Particularly, TmDOTP^5−^ has been infused intravenously to induce ^23^Na compartmental signal separation in healthy^34,40^ and tumor-bearing rats^41^. However, these studies only detected non-localized ^23^Na signals, and thus could not differentiate between Na^+^ across tissues. Our goal was to investigate ∆Na^+^_mem_ and ∆Na^+^_end_ in brain tumors and normal tissues in 3D using ^23^Na-MRSI with TmDOTP^5-^ at high spatial resolution.

In vitro studies established that the ^23^Na chemical shift is more sensitive to [TmDOTP^5−^] than other biological factors such as changes in pH (i.e., ~ 0.4 difference between tumor and healthy tissue^35^ and/or temperature variations (at most ~ 0.5 °C difference across regions^36^ (Fig. 1b–g); Supplementary: Theory). Upon in vivo administration of TmDOTP^5−^, three ^23^Na peaks were observed, corresponding to Na^+^_b_, Na^+^_e_, and Na^+^_i_. The Na^+^_b_ peak was shifted the most, while the Na^+^_i_ peak remained unshifted. Our in vivo results, consistent with prior studies of tumor cells in vitro^19^, demonstrated a significantly weakened ∆Na^+^_mem_ and strengthened ∆Na^+^_end_ within tumor tissue relative to healthy tissue as consequences of elevated Na^+^_b_ and lowered Na^+^_e_, respectively. Additionally, the ^23^Na vascular results showed patterns similar to traditional vascular imaging by ^1^H-based dynamic contrast-enhanced MRI (^1^H-DCE-MRI)^42^. We describe the nuances of these novel measurements of disrupted Na^+^ homeostasis in cancer and their implications.

## Results

### In vitro studies for mechanistic separation of ^23^Na peaks

The goal of these studies was to separate the total Na^+^ signal (Na^+^_T_) into distinct signals for blood (Na^+^_b_), extracellular (Na^+^_e_), and intracellular (Na^+^_i_) pools (Fig. 1b). The shifting mechanism induced by exogenous TmDOTP^5−^ and endogenous biological factors on the ^23^Na chemical shift in vitro is depicted in Fig. 1c–g. A two-compartment coaxial cylinder NMR tube setup in vitro was used to mimic Na^+^ in extracellular/intracellular pools (Figure S1). The inner (smaller) and outer (larger) compartments both contained 150 mM NaCl while the latter also contained TmDOTP^5-^ at various concentrations. The whole setup was subjected to several different pH and temperature conditions. The inner compartment, which lacked TmDOTP^5−^ to represent the intracellular space in vivo, produced a small but unshifted ^23^Na peak at 0 ppm. The larger ^23^Na peak was shifted downfield by TmDOTP^5−^, with the difference in peak integrals stemming from different compartment volumes (Figure S1). The feasibility of this approach to quantify Na^+^ signals from different compartments is demonstrated by switching the contents of the compartments and then repeating the above measurements (Figure S1).

In vitro ^23^Na spectra revealed that the chemical shift was most sensitive to [TmDOTP^5−^] changes, compared to pH and temperature variations (Fig. 1c). The ^23^Na shiftability for TmDOTP^5−^ (${s}_{[{paraCA}^{n-}]}$=2.77 ppm/mM; Eq. (3) in Supplementary: Theory) was 11.1 × larger than the shiftability for pH (${s}_{pH}$=0.25 ppm/pH unit) and 92.3 × larger than the shiftability for temperature (${s}_{T}$=0.03 ppm/°C). This means that addition of 1.1 mM TmDOTP^5-^ would induce a ~ 3 ppm shift in the ^23^Na peak. Conversely, a maximal change of 0.4 in pH units, which is observed between normal and tumor tissues^35^, would induce only a ~ 0.1 ppm ^23^Na shift. A similar ^23^Na shift by temperature would require a 3.3 °C change, which is unlikely in vivo*.* Based on the pH and temperature ranges observed in vivo (including tumors), the effect from [TmDOTP^5-^] dominates the ^23^Na chemical shift (Eq. (2) in Supplementary: Theory) by 95%. Therefore, [TmDOTP^5-^] is several orders of magnitude more sensitive in shifting the ^23^Na resonance than typical in vivo factors. Furthermore, the ^23^Na shiftability arising from [TmDOTP^5−^] and pH/temperature effects is much larger than *T*_*2*_ broadening from TmDOTP^5−^ (Figs. 1c and S1). Consequently, ^23^Na spectra displayed dependence mostly on [TmDOTP^5−^] (Fig. 1d,e). However for in vivo scenarios the ranges shown for pH (2 full pH units) and temperature (15 °C interval) are overestimated, and where [Na^+^] far exceeds [TmDOTP^5-^] based on prior experiments^37^. In blood and extracellular spaces, [Na^+^] is ~ 30–100 × greater than [TmDOTP^5-^]^36^. This suggests that the *relative amount* of TmDOTP^5-^ (Fig. 1f,g) is the primary factor affecting ^23^Na chemical shift (Eq. (4) in Supplementary: Theory).

### In vivo separation of ^23^Na peaks indicates compartmentalized Na^+^ pools

**Figure 2:**
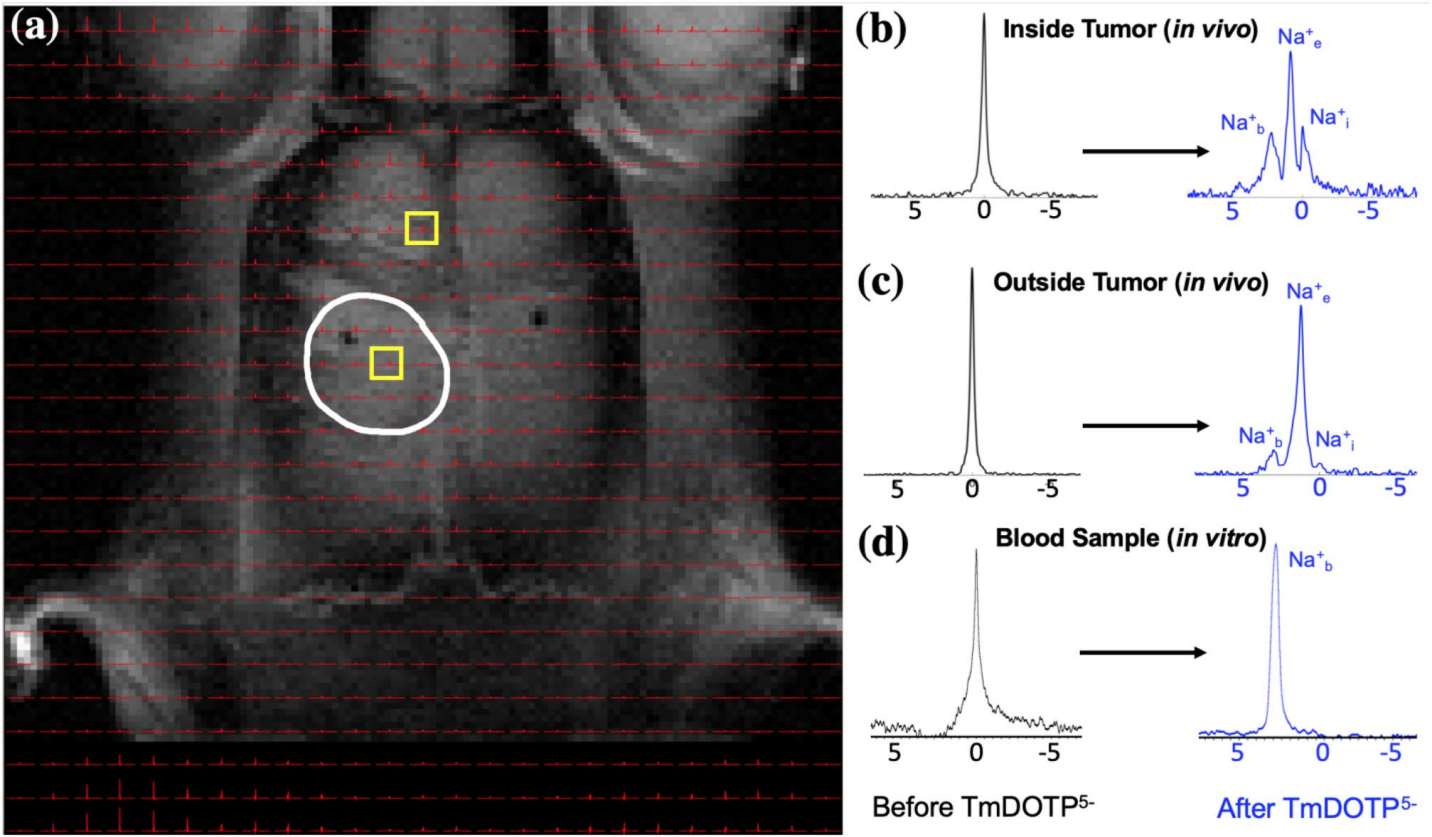
Demonstration of ^23^Na peak separation in vivo following TmDOTP^5−^ administration into a rat bearing a U251 tumor in the brain. (**a**) ^1^H-MRI of an axial slice displaying the anatomical tumor boundary (white outline). The ^23^Na-MRSI is overlaid on top of the ^1^H-MRI. Candidate voxels (**b**) inside and (**c**) outside the tumor are indicated (yellow boxes). Before TmDOTP^5−^ delivery, a single ^23^Na peak was observed at 0 ppm, corresponding to total sodium (Na^+^_T_), both inside and outside the tumor (black spectra). Following TmDOTP^5−^ delivery, compartmental peak separation was achieved to varying extents throughout the brain (blue spectra). (**b**) Within the tumor, this separation was most pronounced due to a compromised blood–brain barrier (BBB), which permits substantial accumulation of TmDOTP^5−^ in the extracellular space. (**c**) Outside of the tumor, such a high degree of extravasation would not be possible, but some shifting is still observed. The TmDOTP^5−^ distribution in the brain warrants labeling the most shifted peak as blood sodium (Na^+^_b_), which occurred consistently around 2 ppm. The unshifted peak, which has no access to TmDOTP^5−^, is intracellular sodium (Na^+^_i_). The intermediate peak, therefore, is extracellular sodium (Na^+^_e_), which is shifted more inside the tumor than outside in healthy tissue. Similar spectroscopic patterns are observed throughout all voxels in vivo. See Figure S2 for a slice below the present. (**d**) In vitro analysis of blood samples from the tumor-bearing rat show that the ^23^Na blood peak occurred around 2 ppm, which coincided with the most-shifted peak we observed in tumor voxels. This confirmed that the most-shifted peak in the observed ^23^Na spectra after TmDOTP^5-^ comes from blood. All spectra were magnitude-corrected and line-broadened by 10 Hz.

Interrogating individual voxels in the brain before and after TmDOTP^5−^ administration (~ 1 µmol/g body weight (BW)) revealed clear ^23^Na signal separation, although to varying extents depending on the degree of TmDOTP^5-^ extravasation from blood to the extracellular space. ^23^Na-MRSI data overlaid on ^1^H-MRI anatomy of rat brains bearing U251 tumors showed spectra in tumor and healthy tissue voxels (Fig. 2a), with candidate voxels inside (Fig. 2b) and outside (Fig. 2c) the tumor before and after TmDOTP^5−^. Before TmDOTP^5-^ delivery, there was a single ^23^Na peak at 0 ppm corresponding to Na^+^_T_, observed ubiquitously both inside and outside the tumor. Upon TmDOTP^5-^ delivery, compartmental ^23^Na peak separation was achieved. Within the tumor, the compromised BBB permitted greater TmDOTP^5−^ extravasation and accumulation in the extracellular space, explicitly yielding three separate ^23^Na peaks emerging from the original single ^23^Na resonance. Each peak was associated with a compartment, with Na^+^_i_ being the unshifted peak (0 ppm) because TmDOTP^5−^ could not enter the intracellular compartment, and other peaks exhibiting [TmDOTP^5-^]-dependent shifts. In the tumor, the most-shifted peak was Na^+^_b_ because the blood compartment had the largest [TmDOTP^5−^], which was corroborated by removing blood samples from the animal and observing the same chemical shift in vitro (Fig. 2d). In the tumor, the intermediate peak in the middle corresponded to the extracellular Na^+^_e_ resonance (Fig. 2b). The splitting was also evident outside of the tumor (i.e., in healthy tissue) where TmDOTP^5-^ extravasated to a much lesser extent compared to tumor tissue (Fig. 2c). The Na^+^_b_ peak was still most-shifted, whereas the Na^+^_i_ and Na^+^_e_ peaks were less discernible. When there was significant overlap between adjacent peaks, instead of peak integrals we used the peak amplitudes to discriminate signals. The shifted bulk Na^+^_e_ peak in healthy tissue confirmed that whatever degree of TmDOTP^5-^ extravasation occurred was sufficient to affect the extracellular ^23^Na signals, albeit less pronounced than tumoral Na^+^_e_. The unshifted Na^+^_i_ resonance was still at 0 ppm, but partially eclipsed by the bulk Na^+^_e_ peak. These same patterns inside/outside the tumor were observed throughout the brain (see Figure S2 for other voxels in the same rat brain).

**Figure 3:**
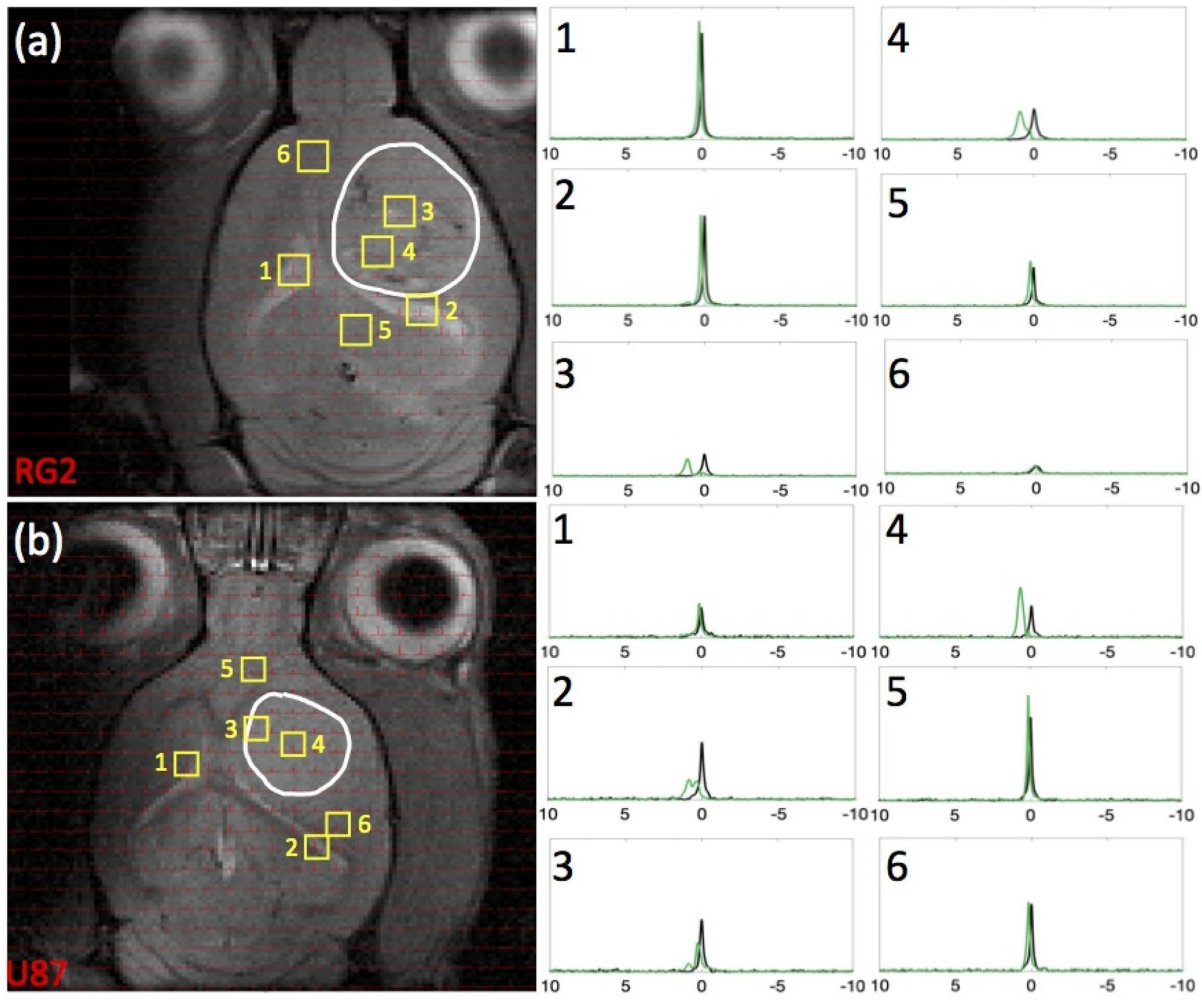
Comparison of ^23^Na peak separation in rats bearing RG2 and U87 tumors. For rats bearing an (**a**) RG2 and (**b**) U87 tumor, the tumor boundary is outlined in white, with voxels of interest indicated in yellow squares (with numbers), and spectra acquired before and after TmDOTP^5−^ delivery shown in black and green, respectively. Tumor voxels 3 and 4 in (**a**) RG2 and (**b**) U87 tumor rats exhibited a fair amount of peak separation due to the leaky BBB. Na^+^_b_ shift was consistently around 2 ppm, and Na^+^_i_ shift was at 0 ppm, whereas Na^+^_e_ shift in the tumor was in the range 0.5–1 ppm. Healthy tissue voxels 5 and 6 in (**a**) RG2 and (**b**) U87 tumor rats were slightly shifted in the downfield direction, suggesting the paramagnetic effects of TmDOTP^5−^ reach the extracellular space even with limited extravasation. Ventricular voxels 1 and 2 in (**a**) RG2 and (**b**) U87 tumor rats displayed a single unshifted Lorentzian peak before and a shifted Lorentzian peak after TmDOTP^5−^ injection. This is attributed to the dominant ^23^Na signal contribution in the ventricles coming from cerebrospinal fluid (CSF), which contains free (i.e., unbound) aqueous Na^+^. The position of the shifted ventricle peak coincided with the Na^+^_e_ peak position in other regions of the brain. This agrees with expectation because CSF is in physical contact with the extracellular space with free exchange of aqueous Na^+^ between the two compartments. Similar spectroscopic patterns are observed throughout all voxels in vivo. See Figure S3 for several slices for each rat shown here. All spectra were magnitude-corrected and line-broadened by 10 Hz.

Figure 3 displays data from representative rats bearing (a) RG2 and (b) U87 tumors, with the array of ^23^Na-MRSI data overlaid on top of the ^1^H-MRI anatomy. The spectra from individual voxels placed throughout the brain confirmed only one ^23^Na peak prior to infusion (Fig. 3, black spectra), corresponding to Na^+^_T_, but upon TmDOTP^5-^ infusion the single peak separated into two additional ^23^Na peaks (Fig. 3, green spectra).

Prior to TmDOTP^5-^ infusion (Fig. 3, black spectra) ventricular voxels [1 and 2 in Fig. 3a,b] exhibited predominantly Lorentzian lineshapes characterized by a single *T*_2_, while those in the normal brain [5 and 6 in Fig. 3a,b] and tumor [3 and 4 in Fig. 3a,b] displayed super-Lorentzian lineshapes indicative of multiple *T*_2_ values. This was because the ventricles are comprised almost entirely of cerebrospinal fluid (CSF), in which all Na^+^ ions are in aqueous media, whereas some Na^+^ ions in tissue can be bound. These observations agreed with prior ^23^Na-MRI results^43–47^.

Administration of TmDOTP^5−^ resulted in the emergence of multiple ^23^Na peaks (Fig. 3, green spectra), particularly within the tumor. However, the downfield shifts seen in healthy tissue suggested the paramagnetic effects of TmDOTP^5-^ were detectable, albeit not as clear as the tumor tissue. We found the most shifted peak sufficiently far from the other two peaks present and attributed it to only Na^+^_b_, with an integral (${\int }_{-0.25 ppm}^{+0.25 ppm}N{a}_{b}^{+}$) reflecting the blood sodium concentration [Na^+^]_b_. Likewise ${\int }_{-0.15 ppm}^{+0.15 ppm}N{a}_{e}^{+}$ and ${\int }_{-0.1 ppm}^{+0.1 ppm}N{a}_{i}^{+}$ measured [Na^+^]_e_ and [Na^+^]_i_, respectively. Tumor voxels [3 and 4 in Fig. 3a,b] exhibited spectra where the three peaks were most notably present. Thus, the chemical shifts of the Na^+^_b_, Na^+^_e_, and Na^+^_i_ peaks can be respectively placed at the same peak positions. Shifts of this nature were evident throughout the entire depth of the brain for both animals (Figure S3). Ventricular voxels [1 and 2 in Fig. 3a,b)] displayed only one Lorentzian peak shifted to the same extent as Na^+^_e_ in healthy tissue voxels [5 and 6 in Fig. 3a,b)], albeit in the latter with super-Lorentzian lineshape. These shifts coincided because CSF and the extracellular space are physically in contact with unrestricted exchange of aqueous Na^+^. Given the shiftability ${s}_{[{paraCA}^{n-}]}$ is 2.77 ppm/mM measured in vitro (Fig. 1), the tumor vasculature contained no more than 0.7 mM TmDOTP^5−^ based on the range of shifts observed. Since the blood ^23^Na signal experienced the greatest shift (Fig. 2d), the (extracellular) tissue therefore encountered even less TmDOTP^5-^, in agreement with prior observations^37^.

### In vivo depiction of transmembrane and transendothelial Na^+^ gradients

**Figure 4:**
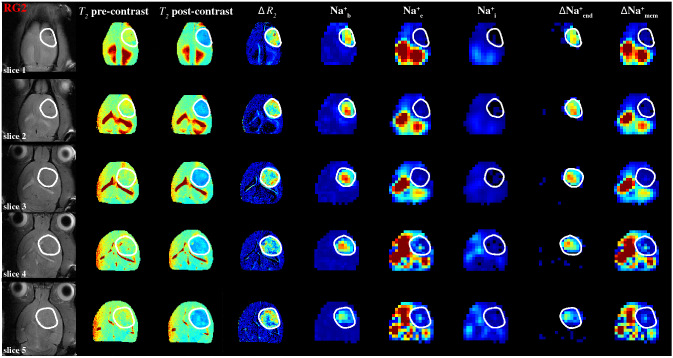
Spatial distributions of compartmentalized ^23^Na signals (Na^+^_b_, Na^+^_e_, Na^+^_i_) as well as transendothelial (ΔNa^+^_end_) and transmembrane (ΔNa^+^_mem_) gradients in an RG2 tumor. The high-resolution ^1^H-MRI data are shown in the left four columns, whereas the lower resolution ^23^Na-MRSI data are shown in the next five columns on the right. The left column shows the tumor location (white outline) on the anatomical ^1^H-MRI (left), whereas the next two columns show the *T*_*2*_ maps (range shown: 0–100 ms) before and after TmDOTP^5−^ injection, and the subsequent column depicts the ∆*R*_*2*_ map (i.e., difference between *1/T*_*2*_ maps before and after, range shown: 0–30 s^−1^), which is proportional to [TmDOTP^5-^] in healthy and tumor tissues. Since ∆*R*_*2*_ values are more heterogeneous within the tumor, the ^23^Na-MRSI data are needed to separate the blood and extracellular compartment signals for the tumor. Since the integral of each ^23^Na peak represents the [Na^+^], the respective three columns show the integral maps of Na^+^_b_, Na^+^_e_, and Na^+^_i_ from left to right (i.e., **∫**Na^+^_b_, **∫**Na^+^_e_, **∫**Na^+^_i_). The last two columns on the right show ΔNa^+^_end_ = **∫**Na^+^_b_-**∫**Na^+^_e_ and ΔNa^+^_mem_ = **∫**Na^+^_e_-**∫**Na^+^_i_. The **∫**Na^+^_b_ map reveals low values in healthy tissue compared to tumor tissue, and within the tumor boundary a high degree of heterogeneity. The **∫**Na^+^_e_ map reveals low values in tumor and normal tissues, but within the tumor boundary a small degree of heterogeneity is visible while ventricular voxels show very high values. The **∫**Na^+^_i_ map reveals low values ubiquitously except some ventricular voxels. The ΔNa^+^_end_ map reveals dramatically high values within the tumor only. The ΔNa^+^_end_ was driven primarily by an increase of **∫**Na^+^_b_ inside the tumor and which was more pronounced in superficial regions of the brain compared to deeper slices. The ΔNa^+^_mem_ map shows low values in tumor tissue compared to normal tissue, although ventricular voxels show very high values. The ΔNa^+^_mem_ is driven primarily by decreased **∫**Na^+^_e_ and thus shows similar level of heterogeneity as the **∫**Na^+^_e_ map. All maps use the same color scale and are relative. See Figure S4 for an example for a U87 tumor.

Integration of compartmentalized ^23^Na spectra (Figs. 2, 3 and S2-S3) generated spatial maps which depicted relative [Na^+^] in each compartment from which the transmembrane (∆Na^+^_mem_ = ∫Na^+^_e_—∫Na^+^_i_) and transendothelial (∆Na^+^_end_ = ∫Na^+^_b_—∫Na^+^_e_) gradient maps could also be calculated, as shown in Fig. 4 for multiple axial slices from a rat brain bearing an RG2 tumor. This 3D high-resolution demonstration of the in vivo Na^+^ biodistribution divulged spatial heterogeneity, where the relative [Na^+^] of each compartment is a function of the compartment volume and the amount of Na^+^ in that compartment. It should be noted that TmDOTP^5-^ extravasation under current conditions was sufficient to observe paramagnetic effects even in healthy brain tissue^38^, and as shown in the *T*_*2*_ maps and concomitant ∆*R*_*2*_ map (given by the difference between inverse *T*_*2*_ maps obtained after and before TmDOTP^5-^ infusion) for RG2 (Fig. 4) and U87 (Figure S4) tumors. While ∆*R*_*2*_ can generally depict the difference between [TmDOTP^5-^] in healthy and tumor tissues^58^, it cannot separate [TmDOTP^5-^] in blood and extracellular compartments within the tumor. Therefore the ∆*R*_*2*_ map cannot fully quantify these separately, while ^23^Na-MRSI spectra can separate these two signals.

There was markedly increased ∫Na^+^_b_ in the tumor, which was not observed elsewhere in normal brain. There was also high degree of heterogeneity within the tumor. The ∫Na^+^_e_ map revealed the largest values in the ventricles (CSF) and smaller values in the tumor with a slight extent of heterogeneity. Outside the tumor, the bulk peak occurred in the integration band for Na^+^_e_. The ∫Na^+^_i_ map unsurprisingly showed values that were about one order of magnitude lower throughout the brain compared to the ∫Na^+^_b_ and ∫Na^+^_e_ maps, since [Na^+^]_i_ (~ 10 mM) is an order of magnitude smaller than [Na^+^]_b_ and [Na^+^]_e_ (~ 150 mM). Furthermore, the ∫Na^+^_i_ values were not significantly different between the tumor and healthy tissue.

**Figure 5:**
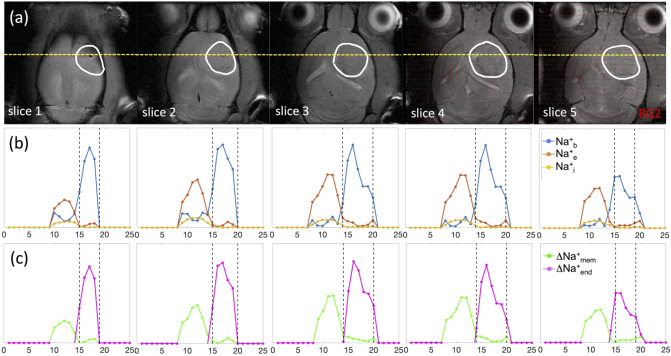
Coronal projections of compartmentalized ^23^Na signals (Na^+^_b_, Na^+^_e_, Na^+^_i_) as well as transendothelial (ΔNa^+^_end_) and transmembrane (ΔNa^+^_mem_) gradients in an RG2 tumor. (**a**) Axial ^1^H-MRI indicating the tumor (white outline) across slices (same as Fig. 4), where the yellow line indicates the position for a coronal projection. (**b**) Spatially varying ^23^Na signals for Na^+^_b_, Na^+^_e_, and Na^+^_i_ are shown with blue, orange, and yellow lines, respectively, where the vertical black lines indicate the tumor boundary. The Na^+^_b_ signal (blue) is clearly elevated in the tumor, and most elevated in slices 1–4 (or superficially). Behavior of Na^+^_b_ signal (blue) is inversely related to Na^+^_e_ signal (orange), which is high outside the tumor and weaker inside the tumor. While intratumoral Na^+^_b_ signal (blue) is high in slices 1–4, the peritumoral Na^+^_e_ signal (orange) is highest in slices 3–4. Comparatively, the Na^+^_i_ signal (yellow) does not vary significantly across slices, but slightly lower inside the tumor than outside the tumor. (**c**) Behaviors of ∆Na^+^_mem_ (green) and ∆Na^+^_end_ (magenta) signals closely mimic patterns of Na^+^_e_ and Na^+^_b_ signals, respectively, indicating that each of those Na^+^ compartments is the primary driver of the respective Na^+^ gradient. See Figure S5 for a similar example for a U87 tumor.

The ∆Na^+^_mem_ values in the tumor were significantly lower compared to the healthy tissue (*p* < 0.05) and the map displayed a similar level of heterogeneity as the ∫Na^+^_e_ map, suggesting that ∆Na^+^_mem_ is driven primarily by the decrease in Na^+^_e_. Ventricular voxels still showed high values in ∆Na^+^_mem_, indicating the large magnitude of Na^+^_e_ in CSF. Likewise, the significant elevation of ∆Na^+^_end_ in the tumor was driven primarily by the Na^+^_b_ increase, and ∆Na^+^_end_ values were significantly larger in the tumor compared to healthy tissue (*p* < 0.05). This feature was more pronounced in superficial regions of the brain because draining veins run vertically down from cortical surface to white matter and some of these blood vessels are perpendicular to the main static magnetic field, thereby enhancing bulk magnetic susceptibility effect on surrounding tissue, which has been reported *in vitro*^31^. This phenomenon is similar to blood oxygenation level-dependent functional MRI contrast mechanisms^48–50^. For both ∆Na^+^_mem_ and ∆Na^+^_end_ gradients, statistical significance was achieved even after excluding ventricle values. These patterns could also be visualized by looking at slice projections of the compartmental and gradient values for the same RG2-bearing animal along a constant coronal position (Fig. 5). For the RG2 tumor, the tumoral increases in Na^+^_b_ and ∆Na^+^_end_ were highest superficially (slices 1–4). Conversely, peritumoral values of Na^+^_e_ and ∆Na^+^_mem_ increased with depth up to a point in the middle of the brain (slices 3–4) before diminishing. Intratumoral Na^+^_e_, however, did not vary significantly with depth. Na^+^_i_ also decreased inside the tumor but not significantly. The ∆Na^+^_mem_ and ∆Na^+^_end_ respectively behaved similarly to Na^+^_b_ and Na^+^_e_ since they were the primary drivers of those gradients. Similar observations were made for U87 tumors (Figures S4-S5) regarding Na^+^ in each compartment and the corresponding gradients.

**Figure 6:**
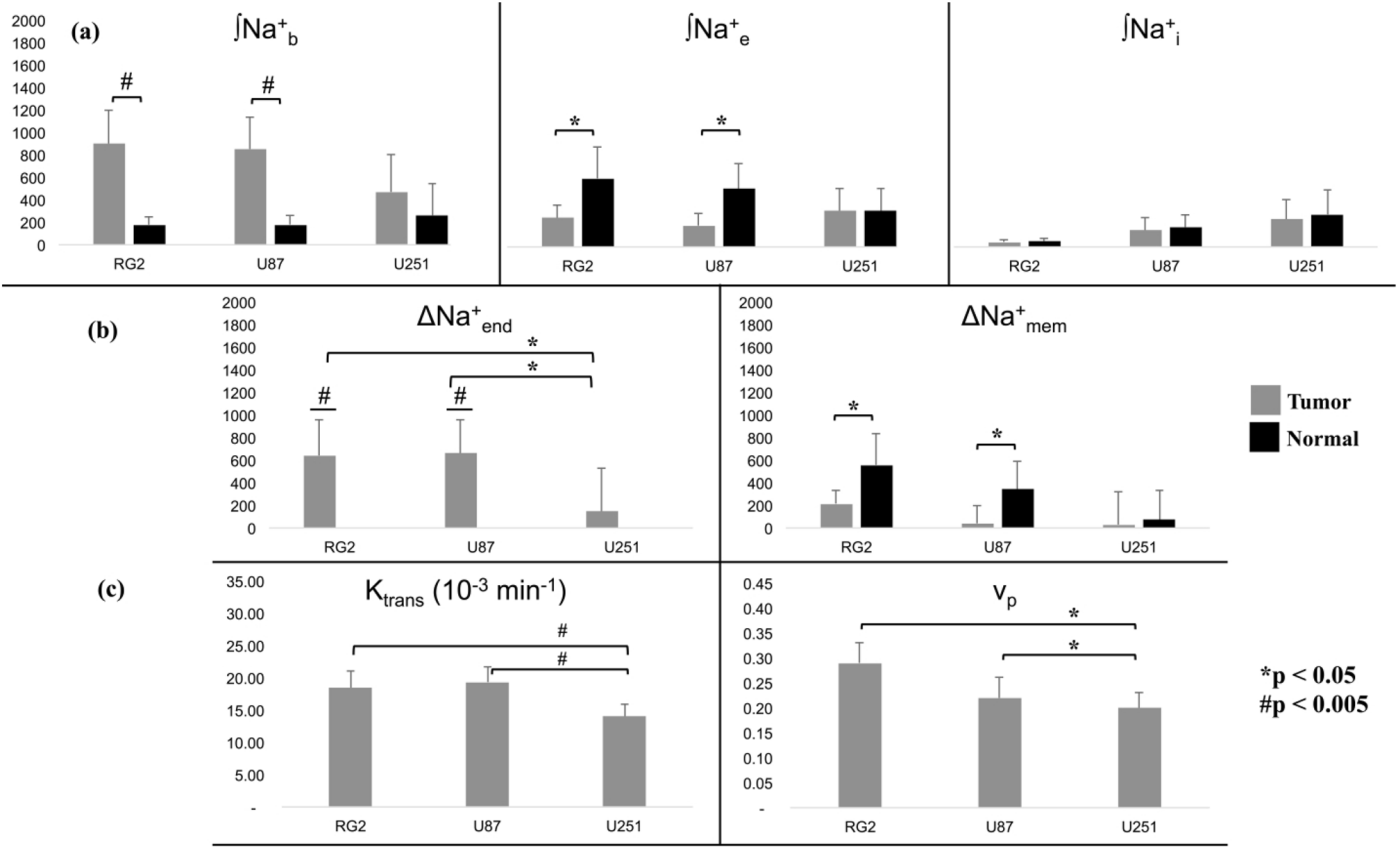
Statistical comparisons between intracellular, extracellular, and vascular compartments across RG2, U87, and U251 tumors with ^23^Na-MRSI and ^1^H-DCE-MRI. (**a**) Relation between ∫Na^+^_b_, ∫Na^+^_e_ and ∫Na^+^_i_ across tumor and healthy tissues. For the RG2 and U87 tumors, the ∫Na^+^_b_ values were significantly higher than normal tissue (*p* < 0.005, #). Also for these tumors, the ∫Na^+^_e_ values were significantly lower than normal tissue (*p* < 0.05, *). The mean values for the U251 tumor roughly followed the same trend but were not significant. Furthermore, there was no significant difference between ∫Na^+^_i_ values in tumor and normal tissues for any of the three tumor types. (**b**) Relations between tumor and normal tissues for ∆Na^+^_end_ and ∆Na^+^_mem_ for the three tumor types. Tumor ∆Na^+^_end_ values were significantly larger than normal values (*p* < 0.005, #), which were non-positive (data not shown). Moreover, ∆Na^+^_end_ in RG2 and U87 tumors was significantly greater than in the U251 tumor (*p* < 0.05, *), indicative of vascular differences between the tumor types. ∆Na^+^_mem_ values were, on average, weaker in tumor compared to normal tissue, but significant only in RG2 and U87 tumors (*p* < 0.05, *). Based on Figs. 5 and S5, it is clear that the relation between ∆Na^+^_end_ and ∆Na^+^_mem_ is negative. (**c**) ^1^H-DCE-MRI data for the volume transfer coefficient (*K*^*trans*^) and plasma volume fraction (*v*_*p*_) values, which are known to reveal information regarding vascular structure and function. *K*^*trans*^ follows the same patterns as ∫Na^+^_b_ and ∆Na^+^_end_ across tumor types. *K*^*trans*^ (*p* < 0.005, #) and *v*_*p*_ (*p* < 0.05, *) were both significantly larger in RG2 and U87 tumors, compared to U251. See Figure S6 for the plasma flow rate (*F*_*p*_) and the extracellular volume fraction (*v*_*e*_) ^1^H-DCE-MRI parameters for each tumor type. See Figure S7 for exemplary maps of ^1^H-DCE-MRI parameters for individual animals from each tumor type.

Throughout the entire cohort of rats (Fig. 6a,b), the mean ∫Na^+^_b_ values were larger and mean ∫Na^+^_e_ values were lower in the tumor compared to normal tissue. These trends were significant in RG2 (*p* < 0.005) and U87 (*p* < 0.05) tumors while there was no significant difference in ∫Na^+^_i_ for all three tumors (Fig. 6a). Identical trends were also observed in ∆Na^+^_end_ and ∆Na^+^_mem_, and significantly so in RG2 (*p* < 0.005) and U87 (*p* < 0.05) tumors. Moreover, ∆Na^+^_end_ was significantly stronger in RG2 and U87 tumors compared to U251 (*p* < 0.05) (Fig. 6b).

Since a strengthened ∆Na^+^_end_ is indicative of impaired vascular integrity, we employed ^1^H-DCE-MRI to reliably image vascular function within the tumor core^42^. Of the four parameters which can be obtained by fitting ^1^H-DCE-MRI data from a two-compartment exchange model, the volume transfer constant (*K*^*trans*^) and plasma volume fraction (*v*_*p*_), as shown in Fig. 6c, both followed the trends of ∆Na^+^_end_ across tumor types: in RG2 and U87 tumors compared to U251, there was a significant difference (*K*^*trans*^: *p* < 0.005 and *v*_*p*_: *p* < 0.05; for plasma flow rate (*F*_*p*_) and extracellular volume fraction (*v*_*e*_) see Figure S6). Although significance was marginal for *F*_*p*_, the mean values followed suit (Figure S6). The ^1^H-DCE-MRI data displayed regions of low *F*_*p*_ and larger *v*_*e*_ within an exemplary slice of a U251 tumor, indicative of a necrotic core, which RG2 and U87 animals lacked (Figure S7). Reduced ∆Na^+^_end_ in U251 tumor (Fig. 6b) could be a marker of necrosis presence as evidenced by lower *K*^*trans*^ and higher *v*_*p*_ in the core of U251 tumors. These results suggest that U251 tumors would have lower tissue and blood oxygenation compared to RG2 and U87 tumors. Additionally, beyond the core of U251 tumors *v*_*e*_ on average was smaller than *v*_*p*_, indicating a high degree of tumor angiogenesis. These findings further substantiate the ∫Na^+^_b_ and ∆Na^+^_end_ results derived from the ^23^Na-MRSI studies. Given *v*_*e*_ and *v*_*p*_ heterogeneity, we attribute the minor ^23^Na peaks downfield of 2 ppm to agent pooling from ineffective agent clearance.

**Figure 7:**
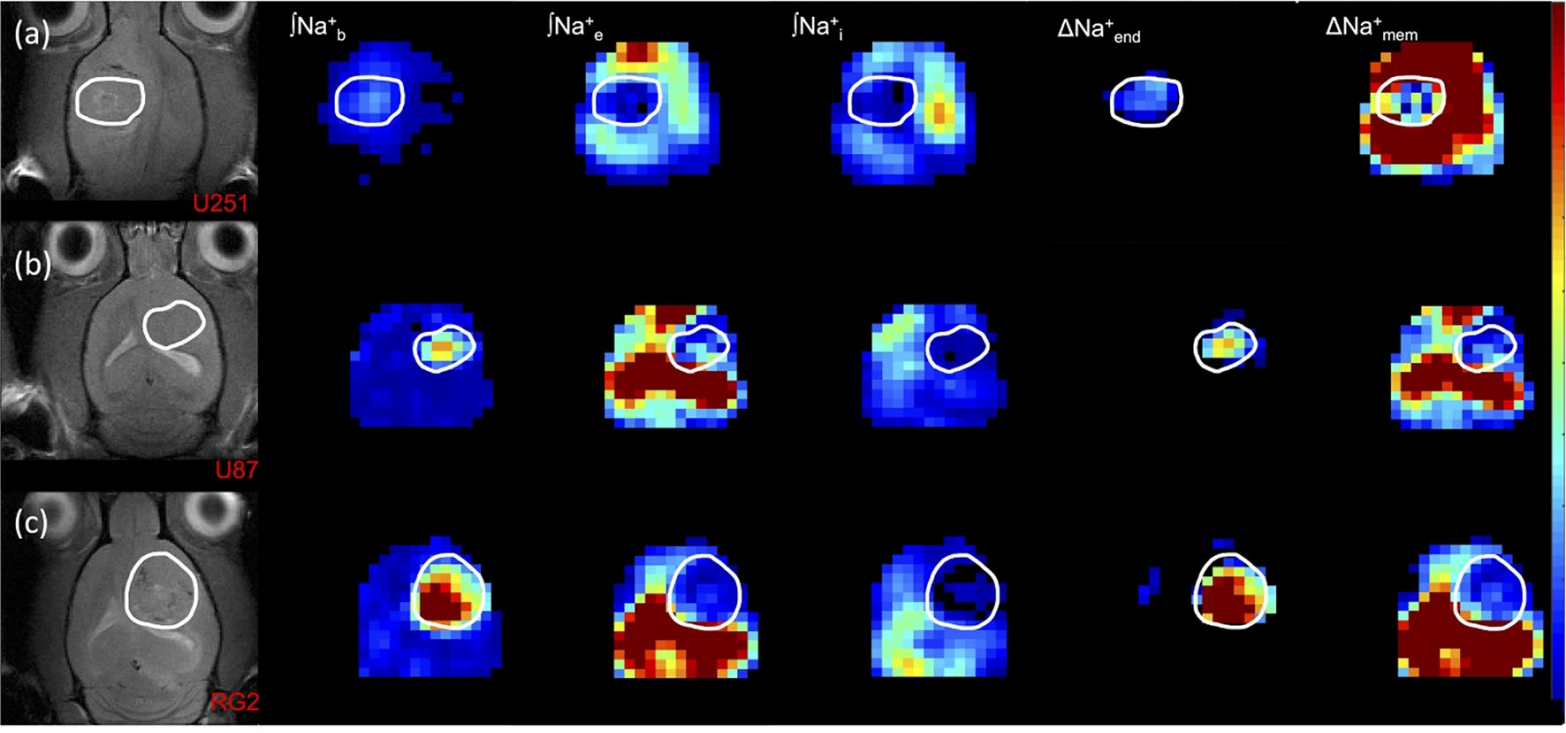
Representative maps of compartmentalized ^23^Na signals (Na^+^_b_, Na^+^_e_, Na^+^_i_) as well as transendothelial (ΔNa^+^_end_) and transmembrane (ΔNa^+^_mem_) gradients in U251, U87, and RG2 tumors. The left column shows the tumor location (white outline) on the anatomical ^1^H-MRI for animals bearing (**a**) U251, (**b**) U87 and (**c**) RG2 tumors. The respective three columns show **∫**Na^+^_b_, **∫**Na^+^_e_, and **∫**Na^+^_i_ maps. The last two columns on the right show the ΔNa^+^_end_ and ΔNa^+^_mem_ maps. In all tumors the **∫**Na^+^_b_ and **∫**Na^+^_e_ are high and low, respectively, and thus are the main drivers for a high ΔNa^+^_end_ and a low ΔNa^+^_mem_.

Figure 7 shows ∫Na^+^_b_, ∫Na^+^_e_, ∫Na^+^_i_, ∆Na^+^_end_, and ∆Na^+^_mem_ maps across all tumor cell lines (RG2, U87, U251). The trends seen previously pervaded all animals, but to varying degrees based on the tumor type. The ∫Na^+^_b_ elevation, and concomitant ∆Na^+^_end_ strengthening, were most pronounced for the RG2 tumor, followed by U87 and then U251. Likewise, the decrease in ∫Na^+^_e_ and weakening of ∆Na^+^_mem_ followed the same order for the tumors. In all tumors, Na^+^_b_ and Na^+^_e_ patterns respectively drove the behaviors of ∆Na^+^_end_ and ∆Na^+^_mem_.

## Discussion

### Implications of current findings

Our results enabled comparisons of Na^+^ physiology and distributions among RG2, U87, and U251 gliomas. Both U87 and U251 are human-derived cell lines, whereas RG2 is derived from rat glioma^51,52^. Experimentally the U251 tumor is most heterogeneous, since U251 cells grow erratically and anisotropically compared with RG2 and U87 cells. Additionally the U251 tumor is more invasive and infiltrative than U87^53^, and U251 cells display greater necrosis, expression of hypoxia-inducible factor 1-alpha (HIF1α) and of Ki67, indicating higher rates of proliferation^54^. U251 cells also test positive for glial fibrillary acidic protein (GFAP) and vimentin, and exhibit neovascularization and angiogenesis. U87 cells are also positive for vimentin and exhibit significant angiogenesis but do not develop necrosis. Neither U251 nor U87 exhibits endothelial proliferation, a common hallmark of human-derived GBM lines^53^. The RG2 tumor exhibits invasiveness and induces BBB disruption, producing edema surrounding the tumor where pericytes help promote angiogenesis to increase permeability of the tumor vasculature^55^. These data concur with our findings. We observed that the negative correlation between the transmembrane and transendothelial gradients were strong in the RG2 and U87 lines but weak for U251. The increase of the transendothelial gradient nearly matched the decrease of the transmembrane gradient in U87 tumors, and exceeded in RG2, which matched behavior regarding BBB permeability. Higher density of blood vessels or higher blood volume would explain higher ^23^Na signal but not necessarily higher Na^+^ concentration in the blood. Although the blood vessels are leaky to gadolinium (Gd^3+^) ions, the elevated transendothelial gradient suggests that the BBB is impermeable to Na^+^, which is well known^2^.

Alkylating chemotherapy agents attach an alkyl group to DNA of cancer cells to keep them from replicating. For example, temozolomide (TMZ) achieves cytotoxicity by methylating the O^6^ position of guanine. O^6^-methylguanine-DNA-methyltransferase (MGMT) is a DNA repair enzyme, which ordinarily repairs the naturally occurring DNA lesion O^6^-methylguanine back to guanine and prevents mistakes during DNA replication and transcription. Unfortunately, MGMT can also protect tumor cells by the same process and neutralize the cytotoxic effects of agents like TMZ. If the *MGMT* gene is silenced by methylation in tumor cells (i.e. MGMT-negative or MGMT-methylated), its DNA repair activity is diminished and the tumor’s sensitivity to chemotherapy is amplified. This suggests that MGMT-positive tumor cells become resistant to chemotherapy, and therefore would possess a depolarized *V*_*m*_ due to its proliferative state.

A recent study demonstrated higher MGMT mRNA expression for RG2 compared to U87^56^. Another study showed that the 50% inhibition concentration (IC^50^) of TMZ for U87 and U251 cells are comparable^57^. Together, these suggest that RG2 is most resistant to chemotherapy presumably due to its augmented proliferative/replicative state, and hence a depolarized *V*_*m*_. These observations partially agree with our results, where RG2 and U87 tumors maintain a depolarized *V*_*m*_ for their proliferative/replicative state to persist.

### Study highlights

In vitro ^23^Na shifts were most dependent on [TmDOTP^5-^] given its high shiftability (${s}_{[{paraCA}^{n-}]}$=2.77 ppm/mM), whereas shiftability due to pH and temperature effects were negligible within physiological ranges (${s}_{pH}$=0.25 ppm/pH unit; ${s}_{T}$=0.03 ppm/°C). The maximum pH difference between glioma and brain tissue is ~ 0.4 pH units^35,38,58,59^ whereas temperature differences of ~ 0.5 °C are extremely unusual in the brain^60–62^. Under these extreme conditions, the respective ^23^Na shift variations caused by pH and temperature would be 0.1 ppm and 0.015 ppm, respectively. Meanwhile, TmDOTP^5-^ can reach in vivo concentrations close to 1–2 mM in blood and interstitial spaces^36,37,63^ which would cause ^23^Na shifts of 2.8–5.5 ppm. While we did not attempt to calculate [TmDOTP^5-^] in each voxel, the sodium shiftability was specifically used to discern that the most shifted peaks were from the blood and extracellular (or CSF) compartments, respectively. Given observed ^23^Na line widths in vivo on the order of ~ 0.4 ppm, TmDOTP^5-^ concentration effects dominate the shifting effect (96–98%). Therefore, ^23^Na shiftability can be considered a univariate function of [TmDOTP^5-^] in vivo.

These observations enabled attributing individual ^23^Na peaks to specific in vivo pools for blood, extracellular and intracellular spaces arising from compartmental differences in [TmDOTP^5-^] upon intravenous administration (~ 1 µmol/g). The ^23^Na shifts in tumor tissue (~ 0.5–1 ppm) were more conspicuous compared to peritumoral tissue, but lower than in blood (~ 2 ppm), suggesting larger [TmDOTP^5-^] in the blood compartment. Additionally, the blood and extracellular peaks were separated by ~ 1.5 ppm, much larger than their line widths (~ 0.4 ppm), indicating minimal cross-compartmental contributions.

Integrating the separated ^23^Na peaks enabled spatial mapping of Na^+^ compartments and gradients in vivo. In the tumor, compared to normal tissue, the transendothelial Na^+^ gradient was stronger and the transmembrane Na^+^ gradient was weaker due to elevated blood and decreased extracellular ^23^Na signals. Current agent concentration does not affect the Na^+^ gradients, simply the chemical shift position of the ^23^Na peak for each compartment. However, an order of magnitude higher agent concentration would affect the estimation of the Na^+^ gradients due to amount of Na^+^ ions co-infused. The enhanced ^23^Na blood signals in tumors complied with dynamic ^1^H-DCE-MRI scans based on Gd^3+^ uptake, which revealed a higher degree of vascularity in RG2 and U87 tumors. Extracellular Na^+^ signal in the ventricles was also very high due to the presence of the agent in CSF^63^. However, ventricular ^23^Na peaks were Lorentzian because CSF contains primarily aqueous Na^+^ and thus a single *T*_*2*_ component, whereas tissue ^23^Na peaks appeared super-Lorentzian because semi-solid Na^+^ binding in tissue resulted in multiple *T*_*2*_ components^64^.

### Comparison with previous work

The present in vitro data improve upon earlier attempts at quantifying ^23^Na shiftability using paraCA^*n-*^ versus many parameters like pH, temperature and other cations^65^. However, the findings focused more on characterizing the dependence on each parameter (linear, sigmoidal, etc.) rather than considering relevant in vivo conditions. Additionally, the model was not employed in the context of the brain/other tissues. Our ^23^Na shiftability model does not require assessing the effects of cationic competition for attraction to TmDOTP^5-^^66^ because other cations are not present in blood and/or extracellular spaces in concentrations comparable to Na^+^^67^.

Prior in vivo ^23^Na-NMR spectroscopy studies utilizing TmDOTP^5-^ in the brain failed to elucidate spatial information, and instead only focused on acquisition of either global data or localized voxels^34,40^. The findings reported two broadened peaks, an unshifted intracellular peak and a shifted extracellular peak. Based on two peaks over limited spatial regions, these studies could not comment specifically on the spatial distribution of transmembrane gradient. Furthermore, the blood ^23^Na signal was not separated so the transendothelial gradient could not be assessed. The shifting capability of TmDOTP^5-^ for separating ^23^Na resonances in tumor tissue was demonstrated in situ, but still at a global level and without mention of Na^+^_b_ specifically^41,68^.

Recently, ^23^Na-MRI methods have been preferred clinically over spectroscopic methods^26^. Such relaxometric modalities exploit differences in diffusion and relaxation behavior between Na^+^ ions inside/outside the cell, because intracellular ions are generally considered less mobile due to binding. Due to the spin-3/2 of ^23^Na, this binding amplifies the relative contribution of nuclear satellite transitions and permits the use of MQF techniques to isolate signals from individual in vivo compartments. However, these ^23^Na-MRI methods are currently limited for probing intracellular Na^+^ because they fail to completely suppress ^23^Na signals from the blood and extracellular compartments^26^. While ^23^Na-MRI results with MQF or ADC can be compared with ^23^Na-MRSI results, duration of all experimental measurements will require several hours and may put strain on animal’s physiology. However future studies can compare MRSI data with ADC and/or MQF data in different groups.

Our method avoids this practical ^23^Na-MRI limitation to provide physiological information about all relevant aqueous compartments, and thus enables estimation of transendothelial and transmembrane gradients. Overall, the ^23^Na-MRSI results agree with prior findings that a depolarized *V*_*m*_ (i.e., weakened transmembrane gradient) is responsible for tumor proliferation^19^. Given that both the cell membrane and BBB help to maintain the ionic level of the extracellular fluid^1,2^, our results also show that the transendothelial gradient is significantly enhanced in the same tumors that show compromised BBB integrity (i.e., RG2 and U87). Together these suggest that the current ^23^Na-MRSI scheme can be used to study the perturbed sodium homeostasis in vivo within the neuropil.

### Technical limitations

This technique, while a crucial first step towards mapping the spatial distribution of Na^+^ in vivo, cannot yet absolutely quantify [Na^+^], although the ratios of the Na^+^_b_, Na^+^_e_, and Na^+^_i_ integrals measured reflect the physiological ratios outside of the tumor, and are altered inside the tumor. Including a quantifiable standard can usually circumvent this limitation because ^23^Na-NMR has no endogenous species that can be used as a standard. However, using the strong CSF signal in vivo remains a possibility for future explorations. Setups involving Na^+^ phantoms with relatively large [TmDOTP^5-^] within the field-of-view (FOV) alongside the body region being imaged could be used, but these could hinder the shim around the subject’s body part being imaged, a technical necessity for the ^23^Na-MRSI method, and these components are difficult to cover with radio frequency (RF) surface coils. Additionally, broad point-spread functions make quantifications in external phantom standards challenging, though they are perhaps the best option presently^69^. Moreover, RF inhomogeneity was not directly addressed in this study. To partially circumvent this shortcoming, we used axial slices (parallel to the plane of the RF coil) to a depth of the brain where sufficient sensitivity was achievable. However, this limitation could be overcome with use of adiabatic RF pulses in the future.

Contrast agents with lanthanide(III) ions (Ln^3+^) are popular in molecular imaging with ^1^H-MRI^13,70^, but clinically the preference is probes with Gd^3+^ conjugated to linear or cyclical chelates^71,72^. The most biocompatible Gd^3+^ chelates are based on 1,4,7,10-tetraazacyclododecane-1,4,7,10-tetraacetate (DOTA^4-^) because they are both kinetically and thermodynamically stable^73^. A LnDOTA^-^ carries a -1 charge. But if a phosphonate group is attached to each of the pendant arms in DOTA^4-^, then DOTP^8-^ is formed and complexation with Ln^3+^ permits a -5 charge (e.g. TmDOTP^5-^). The majority of paraCA^*n-*^ that will work for the type of ^23^Na-MRSI experiments described here are based on Ln^3+^ complexes, because these give rise to large ^1^H hyperfine shifts^74^. But there is growing attention on complexes with similar paramagnetic properties from transition(II) metal ions (Tn^2+^), such as Fe^2+^, Ni^2+^, or Co^2+^^75^. The Tn^2+^-based paraCA^*n-*^ has the potential for clinical use because of superior biocompatibility. Some Tn^2+^ complexes designed could carry a -5 charge, but studies need to explore the safest and most effective paraCA^*n-*^ for ^23^Na-MRSI experiments.

Another limitation is the infusion of a small amount of Na^+^ with the paraCA^*n-*^ itself. TmDOTP^5-^ exists commercially in the form Na_5_TmDOTP, so a small amount of Na^+^ is being added. Since [TmDOTP^5-^] does not exceed 2 mM in the brain vasculature (as determined by the maximum Na^+^_b_ shift), there is at most ~ 1.3% increase of the endogenous [Na^+^] in blood/extracellular spaces. In regions with high [TmDOTP^5-^] and low [Na^+^], like necrotic core of tumors (e.g., U251 tumors), the infused Na^+^ may represent a larger percentage. However, necrotic cores can be identified with *T*_*2*_-weighted ^1^H-MRI scans. Since extracellular Na^+^ is shifted less than blood, it is highly doubtful that enough Na_5_TmDOTP extravasation occurs to significantly alter the relative Na^+^ levels between compartments and impact the conclusions drawn from this study. Future studies with selective BBB opening using focused ultrasound can resolve the uncertainties if intracellular Na^+^ is lower or higher in tumor compared to normal tissue. Furthermore, we are confident that this additionally infused Na^+^ does not enter the intracellular compartment and confound the results. Studies indicate that aggressive cancers express embryonic isoforms of voltage-gated sodium channels^76–78^. Since *V*_*m*_ during the embryonic state is depolarized^19^, this suggests there is little Na^+^ influx from the extracellular compartment.

Another confounding factor is the use of isoflurane during both the repeated ^1^H-MRI tumor monitoring scans, and the ^23^Na-MRSI imaging session. The use of anesthesia is necessary to reduce movement errors in awake animal MRI/MRSI experiments, but all precautions should be taken to reduce the period of anesthesia exposure. However accuracy of this ^23^Na-MRSI method is partly dependent on accurate estimation of [TmDOTP^5-^] per voxel. While *T*_*2*_ maps before and after TmDOTP^5-^ injection can partly estimate [TmDOTP^5-^] in healthy and tumor tissues, this ^1^H-MRI approach cannot separate [TmDOTP^5-^] in blood and extracellular compartments within the tumor. However future spatial resolution improvements of ^23^Na-MRSI in relation to ^1^H-MRI could enable this approach to be implemented better for improved estimates of [TmDOTP^5-^] in blood and extracellular compartments within each voxel.

## Conclusion

This study is the first to image the transformed transmembrane and transendothelial gradients of gliomas using TmDOTP^5−^ for 3D ^23^Na-MRSI at high spatial resolution (1 µL/voxel). The in vivo data consistently revealed weakening transmembrane gradient and strengthening transendothelial gradient within tumors compared to normal tissue, which partially agree with prior findings^19^ and suggest that tumors experience a redistribution of Na^+^ across compartments. There is good evidence to propose that these measurements could potentially probe stages of the cell cycle (transmembrane gradient), and perhaps, angiogenic behavior (transendothelial gradient). The described ^23^Na-MRSI method could empower testing of novel chemotherapy and anti-angiogenic drugs for GBM models, which even at a preclinical level would be significant. This method could potentially be translated into patients by using Tn^2+^-based or Gd^3+^-based paraCA^*n-*^ such that suitable therapies can be targeted based on MGMT screening in GBM patients.

## Materials and methods

### In vitro characterization

In vitro experiments were performed using a 2-compartment coaxial cylindrical 7-inch NMR tube setup from WilmadLabGlass (Vineland, NJ, USA). One compartment contained 150 mM NaCl and the other contained the same but with varying amounts of TmDOTP^5−^ (1 − 10 mM) and 10% v/v ^2^H_2_O to lock the spectrometer frequency using the ^2^H_2_O signal (Figure S1). NaCl and ^2^H_2_O were purchased from Sigma-Aldrich (St. Louis, MO, USA), and TmDOTP^5−^ was purchased as the sodium salt Na_5_TmDOTP from Macrocyclics (Plano, TX, USA). The 5-mm opening of the NMR tube permitted an insert (the inner compartment) whose 50-mm-long tip had inner and outer diameters of 1.258 and 2.020 mm, respectively. The outer-to-inner volume ratio between the two compartments was 8.6. The geometry of the setup allowed 645 µL total in the outer compartment to fill around the tip. Each solution was pH-adjusted using HCl or NH_4_OH to give 5 different pH values.

^23^Na-NMR spectra were collected on a Bruker Avance III HD 500 MHz vertical-bore spectrometer (Bruker, Billerica, MA, USA) interfaced with Bruker TopSpin v2.1 software. We used a Bruker 500 MHz TBO liquid broadband probe with *z*-gradient in all frequencies and an inner coil was used to detect X-nuclei. A single ^23^Na square pulse (50 µs) was used to globally excite the volume of interest (repetition time *T*_*R*_ = 275 ms) collecting 2048 free induction decay (FID) points in the time domain with an acquisition time *t*_aq_ = 38.9 ms, averaged 4096 times. Each set of scans was repeated at a series of temperatures: 27, 30, 34, 37, and 40 °C. Spectra were analyzed using 10 Hz line broadening (for improved delineation of major peaks and reduction of baseline noise) and manual zeroth- and first-order phasing. Data points were fit to Chebyshev rational polynomials using TableCurve 3D v4.0.05 (Systat Software, San Jose, CA, USA).

### In vivo studies

The in vivo protocol was approved by the Institutional Animal Care & Use Committee of Yale University, and all procedures were performed in accordance with these enforced guidelines and regulations, and in compliance with the ARRIVE guidelines. Rats (athymic/nude and Fischer 344) were purchased through Yale University vendors. U251, U87 and RG2 GBM cell lines were purchased from American Type Culture Collections (Manassas, VA, USA). The U251, U87, and RG2 cells were cultured and grown in a 5% CO_2_ incubator at 37 °C in either low-glucose (U251 cells) or high-glucose (U87 and RG2 cells) Dulbecco’s Modified Eagle’s Medium (DMEM) (Thermo Fisher Scientific, Waltham, MA, USA) with 10% fetal bovine serum (FBS) and 1% penicillin–streptomycin. Cells for tumor inoculation were harvested upon reaching at least 80% confluence and were prepared in FBS-free DMEM. Athymic/nude rats were injected intracranially with 2 − 5 × 10^6^ tumor cells either from the U251 (*n* = 6) or the U87 (*n* = 8) cell line (5-µL aliquot) while placed in a stereotactic holder on a heating pad. Fischer 344 rats were injected with 1.25 × 10^3^ RG2 cells (*n* = 8). During the procedure, animals were anesthetized via isoflurane (Isothesia) inhalation (3 − 4%), purchased from Covetrus (Portland, ME, USA). Dissection was performed to the cranium, and a hole was drilled into the skull to allow for stereotactic needle insertion. Injections were performed using a 10-µL Hamilton syringe with a 26G needle into the right striatum for majority of the experiments, 3 mm to the right of the bregma and 3 mm below the dura. The cells were injected steadily at 1 µL/min over 5 min and the needle was left in place for an additional 5 min post-injection. The syringe was then gradually removed to preclude any backflow of cells. The hole in the skull was sealed with bone wax, and the incision site was sutured after removal of the syringe. Animals were given bupivacaine (2 mg/kg at incision site) and carprofen (5 mg/kg, subcutaneously) during the tumor inoculation to relieve pain. Carprofen was subsequently given once per day for two days post-inoculation.

Rats were weighed daily and kept on a standard diet of rat chow and water. Tumor growth was monitored every 3–4 days using ^1^H-MRI under isoflurane anesthesia. When the tumor had reached a minimum mean diameter of 3 mm at 20–24 days post-injection, each animal was imaged using ^1^H-MRI and ^23^Na-MRSI. An infusion line was first established through cannulation of the tail vein as a means to administer fluids and the paraCA^*n-*^. During the cannulation procedure, the rat was placed on a heating pad to maintain physiological body temperature. A 30G needle, fitted onto a PE-10 line, was inserted into the tail vein while the animal was under anesthesia (also isoflurane 2–3%). The animal was then given Puralube Vet Ointment (Dechra, Overland Park, KS, USA) over the eyes and then situated in a prone position underneath an in-house built ^23^Na/^1^H quad surface coil before being placed in the magnet. The 2.5-cm ^23^Na coil was placed directly on top of the head, and the two 5-cm ^1^H coils flanked the head on the left and right sides. Breathing rate was measured by placement of a respiration pad under the torso, and temperature was monitored through a rectal fiber-optic probe thermometer.

Imaging was conducted on a 9.4 T horizontal-bore Bruker Avance system (Billerica, MA), interfaced with Bruker ParaVision v6.0.1 software running on CentOS. Rats weighed 200–260 g at the time of imaging. Positioning and power optimizations for ^1^H signals were performed using Bruker-defined gradient-echo (GE) and fast spin-echo (FSE) sequences. Shimming was done on the ^1^H coils using an ellipsoid region (12 × 7 × 11 mm^3^) to bring the water linewidth to less than 30 Hz using B_0_ mapping with second-order shim corrections. Pre-contrast ^1^H anatomical MRI was first performed using a spin-echo sequence with 9 axial slices (FOV: 25 × 9 × 25 mm^3^, 128 × 128 in-plane resolution) over 10 echo times *T*_*E*_ (10 − 100 ms) with *T*_*R*_ = 4 s). The multiple echo times enabled voxel-wise calculations of ^1^H *T*_2_ values. ^23^Na power optimizations were then performed using a 2-ms 90° Shinnar-Le Roux (SLR) RF pulse over a 4096 Hz bandwidth (ν_0_^Na^ = 105.9 MHz at 9.4 T), where the optimal 90° pulse power was achieved using less than 8 W.

^23^Na-MRSI in 3D was performed without slice selection using an SLR pulse, where the FOV was 25 × 19 × 25 mm^3^ using a nominal voxel size of 1.0 × 1.0 × 1.0 mm^3^ (i.e., point-spread function was ~ 2.24 × 2.24 × 2.24 mm^3^)^60^, with *T*_*R*_ = 300 ms, and phase encoding (gradient duration = 1 ms, sweep width = 8 kHz, encoding steps = 1027, *k*-space radius factor = 0.55) was done in all three spatial dimensions to avoid chemical shift artifacts caused by slice-selective RF pulses. A preliminary ^23^Na-MRSI scan (same parameters/conditions) was run before administering paraCA^*n-*^. The in vivo ^1^H-MRI delineated the tumor and brain boundary and permitted co-registration with ^23^Na-MRSI data, both before and after infusion of paraCA^*n-*^, enabling anatomical localization of ^23^Na-MRSI spectra at the voxel level. All spectra were line-broadened by 10 Hz and magnitude-corrected.

The animals were then given ~ 1 µL/g BW probenecid using a syringe pump (Harvard Apparatus, Holliston, MA, USA) for 10 min followed by a 20-min waiting period. Then Na_5_TmDOTP (1 µmol/g BW) was co-infused with probenecid (same dose) at a rate of 15 µL/min. This infusion protocol with probenecid has been shown to increase TmDOTP^5-^ retention in vivo without the need for renal ligation, and yielded ~ 0.1 µmol TmDOTP^5-^/g BW in the cortex and subcortex^38^. Moreover, the slow infusion rate allows TmDOTP^5-^ diffusion/clearance into other tissue during the infusion, and therefore TmDOTP^5-^ does not accumulate and remain exclusively in the vasculature. Post-contrast ^23^Na-MRSI was performed 30 min after the start of infusion and repeated subsequently thereafter during the infusion. The imaging session was concluded with post-contrast ^1^H-MRI under identical conditions. Rats were sacrificed following imaging by isoflurane inhalation overdose (5%) while the animal was already unconscious during imaging. Death was ensured by inducing bilateral pneumothorax.

^1^H-MRI and ^23^Na-MRSI results were processed and analyzed using home-written code in MATLAB (MathWorks, Natick, MA, USA). Voxel-wise *T*_2_ values for ^1^H were calculated by fitting MRI voxel intensities versus the series of *T*_*E*_ values to a monoexponential curve *e*^−*TE*/*T*2^. Pre-contrast and post-contrast ^1^H *T*_2_ values were used to qualitatively ascertain the success of paraCA^*n-*^ infusion. 3D ^23^Na-MRSI data were reconstructed using Fourier transformation in all spatial and temporal dimensions after 10-Hz line-broadening. Individual ^23^Na peaks were identified and integrated in MATLAB, guided by the changes observed in *R*_*2*_ maps from the ^1^H-MRI data which is roughly proportional to [TmDOTP^5-^] within each voxel. Due to line broadening induced by TmDOTP^5-^, the integration range was different for each compartment to capture the majority of each peak. Integration bandwidths were chosen based on in vitro results and amount of peak separation. The shift for each ^23^Na peak was based on the amount of TmDOTP^5-^ present in each compartment, i.e., most shifted and broadest for blood compartment compared to the extracellular compartment, whereas the intracellular compartment was unshifted and narrowest. The ∆Na^+^_mem_ values were calculated by subtracting ∫Na^+^_i_ from ∫Na^+^_e_, and ∆Na^+^_end_ by subtracting ∫Na^+^_e_ from ∫Na^+^_b_.

### ^1^H-DCE-MRI studies

To measure vascular parameters [*K*^*trans*^ (volume transfer coefficient, min^-1^), *F*_*p*_ (plasma flow rate, min^-1^), *v*_*e*_ (extracellular volume fraction, unitless), *v*_*p*_ (plasma volume fraction, unitless)] from a two-compartment exchange model (2XCM), ^1^H-DCE-MRI was performed on a subset of RG2 (9.4 T), U87 (11.7 T) and U251 (11.7 T) tumors. ^1^H-DCE-MRI data used a ^1^H volume-transmit (8-cm)/surface-receive (3.5-cm) coil.

Baseline images for *T*_*1*_ mapping were acquired using a rapid acquisition with relaxation enhancement (RARE) sequence with six *T*_*R*_ values (0.4, 0.7, 1, 2, 4, 8 s). Seven 1-mm slices covering the extent of the tumor were chosen and images were acquired with a 25 × 25 mm FOV, 128 × 128 matrix and *T*_*E*_ of 10 ms. ^1^H-DCE-MRI acquisition consisted of a dynamic dual-echo spoiled GE sequence with a temporal resolution of 5 s. Images were acquired with *T*_*R*_ = 39.1 ms, *T*_*E*_ = 2.5/5 ms, flip angle = 15°, and one average. Three central slices of the tumor were chosen with identical positioning, FOV (25 × 25 mm), and matrix (128 × 128) to be co-registered to the *T*_*1*_ data. The sequence was repeated every 5 s over a 22 min period with 0.25 µmol/g gadobutrol (Bayer, AG), a gadolinium (Gd^3+^)-containing contrast agent, injected 2 min after the start of the sequence and then flushed with 100 µL heparinized saline. The multi-*T*_*R*_* T*_*1*_ sequence was then repeated at the end of the ^1^H-DCE-MRI acquisition to serve as a post-Gd^3+^ *T*_*1*_ mapping which was used to delineate tumor boundaries. Quantitative *T*_*1*_ maps were generated by fitting voxel-level data to a monoexponential function in MATLAB.

Measurements from *T*_*1*_-weighted images before Gd^3+^ injection were used to transform time-intensity curves into time-concentration curves after the bolus injection. The region of interest (ROI) was placed inside the tumor area, including the rim, as determined by the region of contrast enhancement/uptake. All analysis, including masking the ROI, was performed in MATLAB using the same home-written code. The arterial input function (AIF) was measured by collecting arterial blood samples at discrete time points post-injection. The raw AIF was fit to a bi-exponential curve with a linear upslope during injection of Gd^3+^. Plasma [Gd^3+^] was derived from the blood [Gd^3+^] using a hematocrit of 0.45. The time resolution and duration interval used downstream in the analysis pipeline were adjusted manually.

The exchange-model parameters were estimated by fitting each voxel using Levenberg–Marquardt regression. Because *K*^*trans*^ fitting often converged on local minima instead of the desired global minimum, multiple starting values were used, ultimately choosing the one with the smallest residual. Other variables were less sensitive to the initial condition so a single starting value sufficed.

### Statistics

All statistical comparisons were performed in MATLAB using a 2-sample Student’s *t*-test (i.e., data passed normality tests with Prism in GraphPad, San Diego, CA) whose null hypothesis claimed there was no difference between the means of the two populations being tested. The populations in our analysis were compartmental and gradient ^23^Na signal values (i.e., means and standard deviations of voxel-wise integrals) between tumor and normal tissue and between cohorts of different tumors. For ^1^H-DCE-MRI studies, the populations were different parameter values between different tumors. In all cases, a significance level of 0.05 was used.

## Supplementary Information

Supplementary Information

## Data Availability

Data supporting the findings of this manuscript are available from the corresponding authors upon request.
