# Publisher Correction: Imaging the transmembrane and transendothelial sodium gradients in gliomas

**DOI:** 10.1038/s41598-021-91581-w

**Published:** 2021-06-08

**Authors:** Muhammad H. Khan, John J. Walsh, Jelena M. Mihailović, Sandeep K. Mishra, Daniel Coman, Fahmeed Hyder

**Affiliations:** 1 Department of Biomedical Engineering, Yale University, N143 TAC (MRRC), 300 Cedar Street, New Haven, CT 06520 USA; 2 Department of Radiology and Biomedical Imaging, Yale University, New Haven, CT 06520 USA

Correction to: *Scientific Reports* https://doi.org/10.1038/s41598-021-85925-9, published online 23 March 2021

The original version of this Article contained an error in Figure 4 where MRI images in the far left column had multiple white circles. The original Figure 4 and accompanying legend appear below.

**Figure 4:**
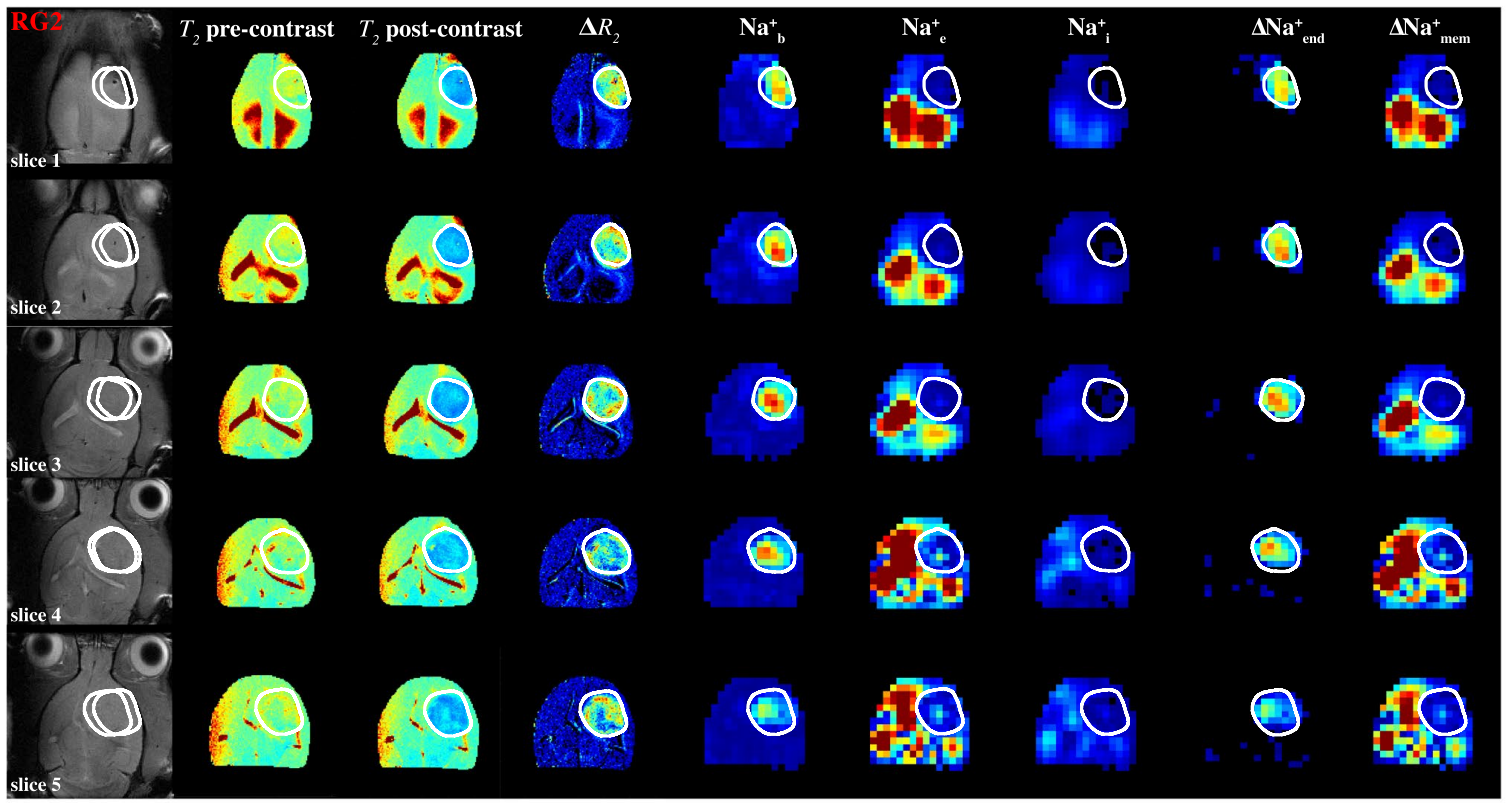
Spatial distributions of compartmentalized ^23^Na signals (Na^+^_b_, Na^+^_e_, Na^+^_i_) as well as transendothelial (ΔNa^+^_end_) and transmembrane (ΔNa^+^_mem_) gradients in an RG2 tumor. The high-resolution ^1^H-MRI data are shown in the left four columns, whereas the lower resolution ^23^Na-MRSI data are shown in the next five columns on the right. The left column shows the tumor location (white outline) on the anatomical ^1^H-MRI (left), whereas the next two columns show the *T*_*2*_ maps (range shown: 0–100 ms) before and after TmDOTP^5−^ injection, and the subsequent column depicts the ∆*R*_*2*_ map (i.e., difference between *1/T*_*2*_ maps before and after, range shown: 0–30 s^−1^), which is proportional to [TmDOTP^5-^] in healthy and tumor tissues. Since ∆*R*_*2*_ values are more heterogeneous within the tumor, the ^23^Na-MRSI data are needed to separate the blood and extracellular compartment signals for the tumor. Since the integral of each ^23^Na peak represents the [Na^+^], the respective three columns show the integral maps of Na^+^_b_, Na^+^_e_, and Na^+^_i_ from left to right (i.e., **∫**Na^+^_b_, **∫**Na^+^_e_, **∫**Na^+^_i_). The last two columns on the right show ΔNa^+^_end_ = **∫**Na^+^_b_-**∫**Na^+^_e_ and ΔNa^+^_mem_ = **∫**Na^+^_e_-**∫**Na^+^_i_. The **∫**Na^+^_b_ map reveals low values in healthy tissue compared to tumor tissue, and within the tumor boundary a high degree of heterogeneity. The **∫**Na^+^_e_ map reveals low values in tumor and normal tissues, but within the tumor boundary a small degree of heterogeneity is visible while ventricular voxels show very high values. The **∫**Na^+^_i_ map reveals low values ubiquitously except some ventricular voxels. The ΔNa^+^_end_ map reveals dramatically high values within the tumor only. The ΔNa^+^_end_ was driven primarily by an increase of **∫**Na^+^_b_ inside the tumor and which was more pronounced in superficial regions of the brain compared to deeper slices. The ΔNa^+^_mem_ map shows low values in tumor tissue compared to normal tissue, although ventricular voxels show very high values. The ΔNa^+^_mem_ is driven primarily by decreased **∫**Na^+^_e_ and thus shows similar level of heterogeneity as the **∫**Na^+^_e_ map. All maps use the same color scale and are relative. See Figure S4 for an example for a U87 tumor.

The original Article has been corrected.

